# *C/N_0_* Estimator Based on the Adaptive Strong Tracking Kalman Filter for GNSS Vector Receivers

**DOI:** 10.3390/s20030739

**Published:** 2020-01-29

**Authors:** Shiming Liu, Sihai Li, Jiangtao Zheng, Qiangwen Fu, Yanhua Yuan

**Affiliations:** 1 School of Automation, Northwestern Polytechnical University, Xi’an 710072, China; liu0749@163.com (S.L.); lisihai@nwpu.edu.cn (S.L.); zheng_183@mail.nwpu.edu.cn (J.Z.); 2 Beijing Institute of Control and Electronic Technology, Beijing 100032, China; yanhuaxiaohua@163.com

**Keywords:** global navigation satellite system (GNSS), vector tracking loops, carrier-to-noise ratio, strong tracking Kalman filter

## Abstract

The carrier-to-noise ratio (*C/N_0_*) is an important indicator of the signal quality of global navigation satellite system receivers. In a vector receiver, estimating *C/N_0_* using a signal amplitude Kalman filter is a typical method. However, the classical Kalman filter (CKF) has a significant estimation delay if the signal power levels change suddenly. In a weak signal environment, it is difficult to estimate the measurement noise for CKF correctly. This article proposes the use of the adaptive strong tracking Kalman filter (ASTKF) to estimate *C/N_0_*. The estimator was evaluated via simulation experiments and a static field test. The results demonstrate that the ASTKF *C/N_0_* estimator can track abrupt variations in *C/N_0_* and the method can estimate the weak signal *C/N_0_* correctly. When *C/N_0_* jumps, the ASTKF estimation method shows a significant advantage over the adaptive Kalman filter (AKF) method in terms of the time delay. Compared with the popular *C/N_0_* algorithms, the narrow-to-wideband power ratio (NWPR) method, and the variance summing method (VSM), the ASTKF *C/N_0_* estimator can adopt a shorter averaging time, which reduces the hysteresis of the estimation results.

## 1. Introduction

Global navigation satellite system (GNSS) receivers use tracking loops to synchronize replicated signals with received signals to maintain a continuous lock on the received signals. Traditional GNSS receivers use scalar-tracking loops (STLs). Vector-tracking loops (VTLs) are a new type of tracking loop. The basic strategy of VTLs is to combine signal tracking and navigation state (position and velocity) estimation into a single algorithm. Compared to STLs, VTLs improve the receiver tracking sensitivity and dynamic adaptability, increase the resistance to interference, and have the ability to bridge short-term interrupt signals [1].

In GNSS receivers, the carrier-to-noise ratio (*C/N_0_*), which is the ratio of the signal power to the noise power density, is an important parameter for measuring the quality of the received signal. In the acquisition phase, *C/N_0_* provides a priori information that is used to determine the optimal detection threshold. In the tracking phase, *C/N_0_* information facilitates the suppression of near-far interference and the avoidance of loss of lock. In particular, various positioning algorithms also use *C/N_0_* to calculate the navigation solutions, such as the weighted least squares method [2]. *C/N_0_* can also be used to facilitate the determination of the current working environment (indoor or outdoor) of the receiver and the construction of an environmentally adaptive navigation system [3]. In a vector receiver, *C/N_0_* is an important basis for determining the validity of the measurements and for estimating the measurement noise [4]. In summary, based on the *C/N_0_*, GNSS receivers can implement a variety of functions to improve performance. The accurate estimation of the *C/N_0_* is vital to developing advanced signal-processing algorithms.

In the GNSS context, various *C/N_0_* estimators have been intensively investigated by a broad range of literature. The main strategy of *C/N_0_* estimation is to construct a statistic that contains the *C/N_0_* information by using the sampled values of the correlator outputs or to directly estimate the signal and noise powers. Commonly used algorithms for *C/N_0_* estimation include the narrow-to-wideband power ratio method (NWPR) [5], the correlator comparison method [5] (the additional noise channel method [6], ANCM), and the variance summing method (VSM) [7]. NWPR is a classic measurement method, which is also known as the standard estimator [8] and is used as a benchmark for algorithm comparison [9]. These estimation algorithms exhibit satisfactory estimation performance in a benign signal environment. However, under weak signal conditions, to obtain an accurate *C/N_0_* measurement, a relatively long averaging time is required, thereby resulting in a significant time lag in the *C/N_0_* estimation [5]. In challenging environments such as dense foliage and urban canyons, the signal power and *C/N_0_* change rapidly if satellite signals are blocked. If the *C/N_0_* shows a large change, the traditional estimation methods must adjust the averaging time, which causes the *C/N_0_* estimation to be delayed, and cannot accurately reflect the change of the real signal.

A fatal drawback of the vector receiver is that a faulty tracking channel can contaminate all of the remaining tracking channels [1,10]. *C/N_0_* is typically an important parameter for the identification of fault-tracking channels [4]. Therefore, timely and accurate estimation of the signal *C/N_0_* is crucial for the vector receiver. The delay in the *C/N_0_* estimation process may cause erroneous measurements to be passed to the navigation Kalman filter (KF), which would severely degrade the navigation performance. For these reasons, the conventional *C/N_0_* estimation algorithms that are discussed above can impair the performance of vector-tracking loops in harsh environments.

In response to possible *C/N_0_* mutations, a *C/N_0_* estimation method that is based on an amplitude Kalman filter is typically used by vector receivers [4,11]. To track rapid changes in the signal amplitude, the ratio of the signal power to the noise power density is monitored [4]. When the signal changes beyond a preset threshold, the covariance matrix of the filter is adjusted. This approach has adaptive processing capability and can track the rapid change of *C/N_0_*. However, the thresholds for detecting signal changes are a predetermined set of fixed values. The thresholds differ among working environments; hence, this method cannot adapt to a wide range of changes in the receiver operating environment. In addition, in a weak signal environment, the amplitude Kalman filter has difficulty accurately determining the measurement noise. Based on this estimation method, an adaptive *C/N_0_* estimation method is presented in [6]. The signal *C/N_0_* is monitored in real time via statistical mutation in a specified time window. The *C/N_0_* estimation algorithm adaptively switches between the differential method and the strong tracking Kalman filter (STKF) estimator. By utilizing the strong tracking filter’s ability to track sudden changes in state, the main drawback of adjusting the covariance matrix according to a preset threshold is avoided. However, the time window statistic for state mutation detection also has a time delay problem, and the measurement noise estimation problem at low *C/N_0_* is not considered.

In this study, we focus on the timely and accurate estimation of *C/N_0_* and the measurement noise statistics in a weak signal environment. This paper proposes a *C/N_0_* estimation method that is based on the strong tracking Kalman filter [12] and the Allan variance-based measurement noise estimation method. The proposed *C/N_0_* estimator avoids the estimation delay that is caused by signal mutation detection and algorithm switching selection. Facilitated by the adaptive estimation of the measurement noise, the estimation accuracy in the weak signal environment is guaranteed. Based on the software receiver, vector-tracking loops and an adaptive *C/N_0_* estimation algorithm are implemented. To evaluate the estimation accuracy and the performance of the algorithm, simulation tests and a static field test were conducted. The adaptive Kalman filter estimator and two popular algorithms (the NWPR method and the VSM method) were selected as references for comparative analysis. The experimental results demonstrate that the *C/N_0_* estimation method that is based on the adaptive strong tracking Kalman filter (ASTKF) has strong advantages in terms of real-time performance and estimation accuracy.

The remainder of this paper is organized in the following manner. The second section describes the basic principles of the vector receiver and the mathematical model that are used in this paper. The algorithmic details of the adaptive *C/N_0_* estimation method are presented in the third section. In the fourth and fifth sections, the proposed method is evaluated via simulation experiments and a static field test. The conclusions of this study are presented in the final section.

## 2. Vector-Tracking Loops

The vector-tracking algorithm is an advanced satellite navigation signal-processing algorithm. A conventional scalar receiver assigns a tracking channel to each visible satellite, and the tracking channel processes the baseband signal using a code loop (delay lock loop, DLL) and a carrier loop (phase lock loop, PLL). Each tracking channel is independent of the others, which ignores the inherent relationships between channels and between navigation parameters and tracking channels [1]. Therefore, this is not a structural form that realizes the best performance. Vector-tracking loops leverage a Kalman filter to simultaneously conduct signal tracking and navigation solutions calculation. The navigation parameters are used to control the operation of the tracking loops, and the correlations between tracking channels are fully considered, which can significantly improve the tracking performance of the receiver. Moreover, the vector-tracking loops are the basis for the construction of a GNSS/inertial deep integration navigation system.

**Figure 1:**
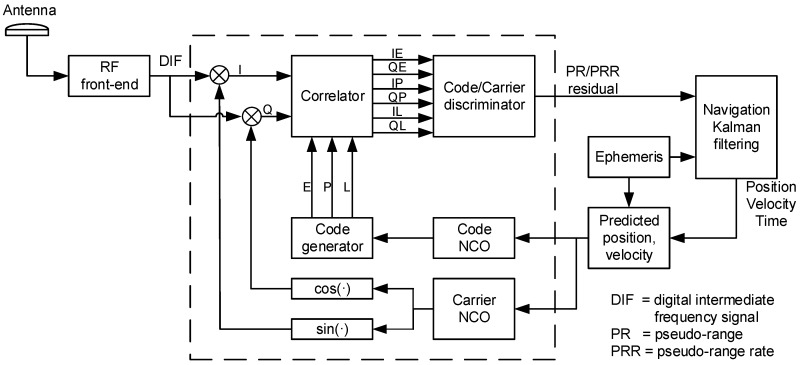
Basic structure of the vector-tracking loops.

The vector receiver uses the traditional acquisition and scalar tracking algorithm to decode the ephemeris and to obtain the first navigation solution, which is used to complete the necessary initialization work [13,14]. In vector mode, the receiver uses the predicted position and velocity and the ephemeris to calculate the control command of the numerically controlled oscillator (NCO) to generate the local replica code and carrier. After the local replica signal and the received signal complete the mixing and correlation operations, the correlation results are input into the code discriminator and the carrier frequency discriminator, and the code phase and carrier frequency tracking error are calculated. Then, the signal-tracking errors are converted into pseudorange residuals and pseudorange rate residuals as the measurement vector of the navigation Kalman filter. States of the Kalman filter are fed back to correct the position and velocity prediction errors. The basic structure of the vector-tracking loops is illustrated in Figure 1. The details of one of the tracking channels are presented in the dashed box.

In Figure 1, the navigation Kalman filtering (NKF) is the core of a vector receiver, which estimates the navigation solutions and closes the tracking loops simultaneously. From the perspective of NKF, the vector-tracking algorithm includes two forms: centralized filtering and federated filtering. The state vector of NKF can select the position-state formulation or the pseudorange-state formulation [15,16]. Centralized filtering avoids the filter divergence that is caused by filter cascade. The position-state formulation has a clearer physical meaning, and the state dimension is lower than the pseudorange-state formulation. Therefore, the centralized filtering structure in the form of the position-state formulation is used as the basic reference frame for vector-tracking loops design.

In the position-state formulation, the typical states of the navigation Kalman filter are the receiver’s position, velocity, and clock states [15]. The state vector of the navigation Kalman filter is

(1) $$X={\left[\begin{array}{cccc} {\left(\delta P_{3\times 1}\right)}^{T} & {\left(\delta V_{3\times 1}\right)}^{T} & {\left(\delta A_{3\times 1}\right)}^{T} & {\left(\delta B_{2\times 1}\right)}^{T} \end{array}\right]}^{T}$$

where $\delta P_{3\times 1}$, $\delta V_{3\times 1}$ and $\delta A_{3\times 1}$ denote the position errors, velocity errors, and acceleration errors, respectively, in three directions and $\delta B_{2\times 1}$ represents the receiver’s clock bias and clock drift errors.

The discretized form of the system equation is as follows:

(2) $$X_{k}=\Phi_{k,k-1}X_{k-1}+\eta_{k-1}$$

where $\Phi_{k,k-1}$ is the state transition matrix, which is formulated as follows:

(3) $$\Phi_{k,k-1}=\left[\begin{array}{cccc} I_{3\times 3} & T_{s}I_{3\times 3} & T_{s}^{2}I_{3\times 3}/2 & 0_{3\times 2}\\ 0_{3\times 3} & I_{3\times 3} & T_{s}I_{3\times 3} & 0_{3\times 2}\\ 0_{2\times 3} & 0_{2\times 3} & I_{3\times 3} & 0_{3\times 2}\\ 0_{2\times 3} & 0_{2\times 3} & 0_{2\times 3} & L \end{array}\right]$$

$$L=\left[\begin{array}{cc} 1 & T_{s}\\ 0 & 1 \end{array}\right]$$

where $T_{s}$ is the discretization interval and $I_{3\times 3}$ denotes a third-order unit matrix.

In Equation (2), $\eta_{k-1}$ is the process noise. The variance matrix is:

(4) $$Q_{k}=\left[\begin{array}{cccc} U\cdot T_{s}^{5}/20 & U\cdot T_{s}^{4}/8 & U\cdot T_{s}^{3}/6 & 0_{3\times 2}\\ U\cdot T_{s}^{4}/8 & U\cdot T_{s}^{3}/3 & U\cdot T_{s}^{2}/2 & 0_{3\times 2}\\ U\cdot T_{s}^{3}/6 & U\cdot T_{s}^{2}/2 & T_{s}\cdot U & 0_{3\times 2}\\ 0_{2\times 3} & 0_{2\times 3} & 0_{2\times 3} & V \end{array}\right]$$

$$U=\left[\begin{array}{ccc} q_{x}^{2} & 0 & 0\\ 0 & q_{y}^{2} & 0\\ 0 & 0 & q_{z}^{2} \end{array}\right],\;V=\left[\begin{array}{cc} q_{b}^{2}T_{s}+q_{d}^{2}\frac{T_{s}^{3}}{3} & q_{d}^{2}\frac{T_{s}^{2}}{2}\\ q_{d}^{2}\frac{T_{s}^{2}}{2} & q_{d}^{2}T_{s} \end{array}\right]$$

where $q_{x}^{2}$, $q_{y}^{2}$ and $q_{z}^{2}$ denote the power spectral density (PSD) of the acceleration disturbance noise in three directions and $q_{b}^{2}$ and $q_{d}^{2}$ are the PSDs of the receiver clock phase noise and the frequency noise, respectively.

The measurement equation of the navigation Kalman filter is as follows:

(5) $$\left[\begin{array}{c} \Delta \rho_{1}\\ \Delta {\dot{\rho }}_{1}\\ \vdots \\ \Delta \rho_{M}\\ \Delta {\dot{\rho }}_{M} \end{array}\right]=\left[\begin{array}{lllll} u_{los,1} & 0_{1\times 3} & 0_{1\times 3} & -1 & 0\\ 0_{1\times 3} & u_{los,1} & 0_{1\times 3} & 0 & -1\\ & & \vdots & & \\ u_{los,M} & 0_{1\times 3} & 0_{1\times 3} & -1 & 0\\ 0_{1\times 3} & u_{los,M} & 0_{1\times 3} & 0 & -1 \end{array}\right]\left[\begin{array}{c} \delta P_{3\times 1}\\ \delta V_{3\times 1}\\ \delta A_{3\times 1}\\ \delta B_{2\times 1} \end{array}\right]$$

where Δρ and $\Delta \dot{\rho }$ are the pseudorange residuals and the pseudorange rate residuals, respectively, that are output by the discriminators; $u_{los}$ denotes the unit vector of the receiver and satellite line of sight; and *M* is the number of visible satellites.

The symbols $IE_{k}$, $QE_{k}$, $IL_{k}$ and $QL_{k}$ represent the early and late complex correlator output samples at time *k*. The correlator spacing is 1/2 chip. $IP_{k,1}$, $IP_{k,2}$, $QP_{k,1}$ and $QP_{k,2}$ represent prompt complex correlator output samples (subscripts 1 and 2 denote the sample outputs in the first half and the second half, respectively, of the integration period). As shown in Figure 1, each tracking channel uses a code discriminator and a carrier frequency discriminator to process the correlator outputs and to estimate the code and carrier tracking errors. The tracking errors of the discriminator output are converted into pseudorange and pseudorange rate residuals as Kalman filter measurements. The forms of the code phase and carrier frequency discriminator are as follows [15].

(1) Code discriminator
  (6) $$Y_{R,k}=\left(IE_{k}^{2}+QE_{k}^{2}\right)-\left(IL_{k}^{2}+QL_{k}^{2}\right)$$
  where $Y_{R,k}$ is the code discriminator output, which has been converted to a pseudo-range residual ${\tilde{Y}}_{R,k}$ (unit: m):
  (7) $${\tilde{Y}}_{R,k}=Y_{R,k}\beta /2A_{F,k}^{2}=\rho_{e,k}+\upsilon_{R,k}\beta /2A_{F,k}^{2}$$
  where $\rho_{e,k}$ is ideal pseudorange residual, β is the chip length (unit: m), $A_{F,k}$ is the signal amplitude, and $\upsilon_{R,k}$ is the discriminator output noise.

Let ${\tilde{\upsilon }}_{R,k}=\upsilon_{R,k}\beta /2A_{F,k}^{2}$. The variance is:

(8) $$\sigma^{2}\left({\tilde{\upsilon }}_{R,k}\right)=\frac{\beta^{2}}{2{\left(Tc/n_{o}\right)}^{2}}+\frac{\beta^{2}}{Tc/n_{o}}\left(\frac{\rho_{e}^{2}}{\beta^{2}}+\frac{1}{4}\right)$$

where T is the predetection integration time and $c/n_{o}$ is the carrier-to-noise ratio in Hz.

(2) Carrier frequency discriminator
  (9) $$\begin{array}{l} Y_{RR,k}=IP_{k,2}QP_{k,1}-IP_{k,1}QP_{k,2}\\ \approx -A_{F,k}^{2}R^{2}\left(\varepsilon_{k}\right)\left(\pi f_{err,k}T\right)/4+\upsilon_{RR,k} \end{array}$$
  where $\varepsilon_{k}$ denotes the code phase error, $R\left(\varepsilon_{k}\right)$ is the C/A code correlation function, $f_{err,k}$ is the frequency error, and $\upsilon_{RR,k}$ is the noise term.

The discriminator output is converted to the pseudo-range rate residual ${\tilde{Y}}_{RR,k}$ (unit: m/s):

(10) $${\tilde{Y}}_{RR,k}={\dot{\rho }}_{err,k}+{\tilde{\upsilon }}_{RR,k}$$

In Equation (10), ${\tilde{\upsilon }}_{RR,k}$ denotes the pseudorange rate residual measurement noise. Its detailed formula and variance are as follows:

(11) $${\tilde{\upsilon }}_{RR,k}=-\frac{4}{A_{F,k}^{2}R^{2}\left(\varepsilon_{k}\right)\pi T}\cdot \frac{c}{f_{L1}}\cdot \upsilon_{RR,k}$$

(12) $$\sigma^{2}\left({\tilde{\upsilon }}_{RR,k}\right)=\frac{2+2R^{2}\left(\varepsilon_{k}\right)\left(Tc/n_{o}\right)}{R^{4}\left(\varepsilon_{k}\right){\left(Tc/n_{o}\right)}^{2}}{\left(\frac{c}{\pi Tf_{L1}}\right)}^{2}$$

where $f_{L1}$ is the Global Positioning System (GPS) L1 frequency and c is the speed of light.

## 3. Adaptive Carrier-to-Noise Ratio (*C/N_0_*) Estimation Method

According to Section 2, the calculation of the noise variances in the vector tracking loops requires signal *C/N_0_* information. In addition, *C/N_0_* can also be used for the detection and isolation of fault-tracking channels.

If there is occlusion of the signal transmission path, the sudden disappearance or occurrence of the signal will cause a sudden change in the signal power, and the *C/N_0_* will also exhibit a short-term large-scale jump. In contrast to conventional receivers, vector receivers can bridge short-term interrupt signals. To fully utilize the advantages of vector-tracking loops, the *C/N_0_* estimation algorithm must adapt to the *C/N_0_* mutation environment. The classical *C/N_0_* estimators have a large delay in response to the sudden change of *C/N_0_*, which will weaken the performance advantage of vector receivers in harsh environments; hence, these estimators are not suitable for vector receivers.

The strong tracking Kalman filter can accurately track the state of a mutation [12]. By using a strong tracking filter to estimate the amplitude of the signal, it can effectively cope with the possible mutation of the received signal. The Allan analysis of variance adaptively estimates the measurement noise by segmenting the frequency bands of the measurement outputs. Based on these two algorithms, a *C/N_0_* estimation method that is based on the adaptive strong tracking Kalman filter is designed.

When the receiver has completed signal acquisition and is tracking a signal well, the coherent integration values of the in-phase (I) and quadraphase (Q) outputs of the prompt branch of the tracking channel can be reduced to functions of $c/n_{0}$ [17].

(13) $$\begin{array}{l} IP_{k}=\sqrt{2\left(c/n_{0}\right)T_{coh}}\sigma_{n}\cos\phi_{e}+n_{I}\\ QP_{k}=\sqrt{2\left(c/n_{0}\right)T_{coh}}\sigma_{n}\sin\phi_{e}+n_{Q} \end{array}$$

where $T_{coh}$ denotes the coherent integration time, $c/n_{0}$ is the carrier-to-noise ratio in Hz, $\phi_{e}$ is the carrier phase error, and $n_{I}$ and $n_{Q}$ are Gaussian white noises with mean zero and variance $\sigma_{n}^{2}$. The coherent integral values are functions of *C/N_0_*. A statistical analysis of the coherent integrated sample values can estimate *C/N_0_*.

Define a statistic in the following form:

(14) $${\tilde{A}}_{k}=IP_{k}^{2}+QP_{k}^{2}$$

Its mean is:

(15) $$E\left\{{\tilde{A}}_{k}\right\}=A_{k}^{2}+2\sigma_{n,k}^{2}$$

(16) $$A_{k}^{2}=2\left(c/n_{0}\right)T_{coh}\sigma_{n,k}^{2}$$

where $A_{k}$ is the effective amplitude of the signal and ${\tilde{A}}_{k}$ is a biased estimate of the square of the signal amplitude.

According to Equation (16), *C/N_0_* in dB-Hz can be expressed as follows:

(17) $${\left(C/N_{0}\right)}_{k}=10\log_{10}\left(\frac{A_{k}^{2}}{2T_{coh}\sigma_{n,k}^{2}}\right)$$

According to Equation (17), estimation of *C/N_0_* requires the signal amplitude and noise variance information. The noise variance is estimated in the receiver via a noise correlator. The noise correlator uses a non-existing pseudo-random noise (PRN) code; hence, its output can be regarded as zero-mean Gaussian white noise [18]. In each 20 ms coherent integration period, the noise correlator outputs *N* samples. Empirical measurements of the noise variance are obtained as:

(18) $${\tilde{\sigma }}_{n,k}^{2}=\frac{1}{N}\sum_{j=1}^{N}n_{j}^{2}$$

where $n_{j}$ represents the noise correlator output.

The interference noise in the receiver tracking channel is mainly thermal noise, and its variance changes slowly with time. Therefore, the noise variance measurement is smoothed to obtain a more accurate measurement [4].

(19) $${\hat{\sigma }}_{n,k}^{2}=\left(1-\alpha \right){\hat{\sigma }}_{n,k-1}^{2}+\alpha {\tilde{\sigma }}_{n,k}^{2}$$

where α is a filter coefficient, the value of which can be found in [4].

Each tracking channel is configured with an amplitude Kalman filter for estimating the signal amplitude. The amplitude Kalman filter is a single-state filter. The discretized system model and measurement equation are as follows:

(20) $$\{\begin{array}{c} X_{k}=X_{k-1}+w_{k-1}\\ Z_{k}=X_{k}+v_{k} \end{array}$$

(21) $$X_{k}=A^{2}+2\sigma_{n}^{2}$$

where A is the effective amplitude of the signal; $Z_{k}$ is the filter measurement, the value of which is equal to $\tilde{A}$ in Equation (14); and w and v are the system noise and the measurement noise, respectively. The state transition matrix $\Phi_{k,k-1}$ and the measurement matrix $H_{k}$ are both 1.

The variance of the measurement noise is:

(22) $$\sigma_{v,k}^{2}=E\left\{{\left[{\tilde{A}}_{k}-E\left({\tilde{A}}_{k}\right)\right]}^{2}\right\}\approx 4{\hat{\sigma }}_{n,k}^{2}\left({\tilde{A}}_{k}-{\hat{\sigma }}_{n,k}^{2}\right)$$

The main reason for the sudden change of the *C/N_0_* due to signal occlusion is the sudden change in the signal power, namely, the signal amplitude (filter state) has a large jump. After the filter reaches a steady state, the classical Kalman filter (see Table 1) state covariance matrix ($P_{k}$) tends to a fixed value. The Kalman filter gain approaches zero. The time update effect is strengthened, and the measurement update function is weakened [19]. This causes the state estimate of the filter to deviate substantially from the true value of the catastrophic state.

To solve the divergence problem of CKF, the strong tracking Kalman filter introduces a time-varying fading factor into the calculation of the state-one-step prediction mean square error matrix, thereby improving the performance in tracking the state. The covariance propagation of the amplitude Kalman filter that is based on the STKF is as follows:

(23) $$P_{k/k-1}=\lambda_{k}\Phi_{k,k-1}P_{k-1}\Phi_{k,k-1}^{T}+Q_{k-1}=\lambda_{k}P_{k-1}+Q_{k-1}$$

where $\lambda_{k}\geq 1$ is a time-varying fading factor.

**Figure 2:**
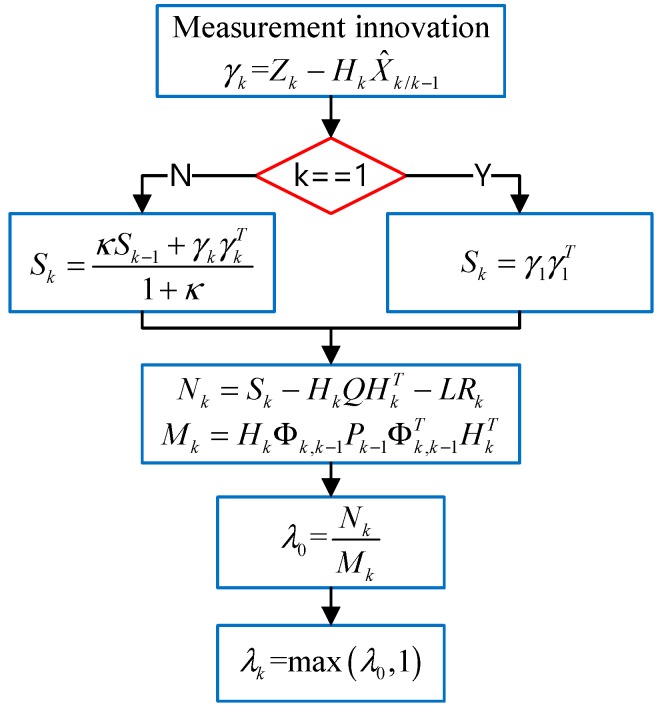
Time-varying fading factor calculation process.

Figure 2 depicts the time-varying fading factor calculation process. In Figure 2, 0<κ≤1 is the forgetting factor and L≥1 is weakening factor.

In the amplitude Kalman filter, the noise variance is estimated via Equation (22). In the case of low *C/N_0_*, $\tilde{A}$ may be smaller than the noise variance ${\hat{\sigma }}_{n}^{2}$. Calculating the noise variance using Equation (22) will result in a negative value, thereby causing the filter to be abnormal. To avoid a negative value of the measurement noise variance, a measurement noise adaptive method that is based on the Allan variance is used to realize the online estimation of the noise variance in real time.

The measurement variance estimation algorithm that is based on the Allan variance is [20]:

(24) $$\begin{array}{l} {\hat{R}}_{k}=\frac{1}{2\left(k-1\right)}\sum_{i=2}^{k}{\left(Z_{i}-Z_{i-1}\right)}^{2}\\ \;\;\;\;=\frac{1}{2\left(k-1\right)}\left[\sum_{i=2}^{k-1}{\left(Z_{i}-Z_{i-1}\right)}^{2}+{\left(Z_{k}-Z_{k-1}\right)}^{2}\right]\\ \;\;\;\;=\left(1-\frac{1}{k-1}\right){\hat{R}}_{k-1}+\frac{1}{2\left(k-1\right)}{\left(Z_{k}-Z_{k-1}\right)}^{2} \end{array}$$

where k=2,3,4,⋯ and the initial value ${\hat{R}}_{1}$ is calculated via Equation (22).

In Equation (24), the estimation process of the measurement noise variance that is based on Allan variance and the Kalman filtering process are completely independent of each other, thereby effectively reducing the risk of Kalman filter divergence [20]. According to Equation (24), the strong tracking filter adjusts the one-step prediction mean square error matrix according to the measurement innovation. Compared with the adaptive method that is based on the innovation sequence, the filtering process that is based on the Allan variance is more stable.

To reduce the influence of the old measurement noise, an exponential fading memory weighted average method can be utilized. The recursive estimation formula for measuring the noise variance is as follows [20]:

(25) $${\hat{R}}_{k}=\left(1-\beta_{k}\right){\hat{R}}_{k-1}+\frac{\beta_{k}}{2}{\left(Z_{k}-Z_{k-1}\right)}^{2}$$

(26) $$\beta_{k}=\frac{\beta_{k-1}}{\beta_{k-1}+b}$$

where the initial value $\beta_{0}=1$ and 0<b<1 is the fading factor.

By combining the noise variance and the state estimate of the amplitude Kalman filter, the following $c/n_{0}$ estimation formula is obtained:

(27) $${\left(\hat{c}/{\hat{n}}_{0}\right)}_{k}=\frac{{\hat{X}}_{k}-2{\hat{\sigma }}_{n,k}^{2}}{2T_{coh}{\hat{\sigma }}_{n,k}^{2}}$$

The *C/N_0_* estimates are averaged over multiple times to obtain more accurate results:

(28) $$\hat{C}/{\hat{N}}_{0}=10\log_{10}\left(\frac{1}{N}\sum_{k=1}^{N}{\left(\hat{c}/{\hat{n}}_{0}\right)}_{k}\right)$$

**Figure 3:**
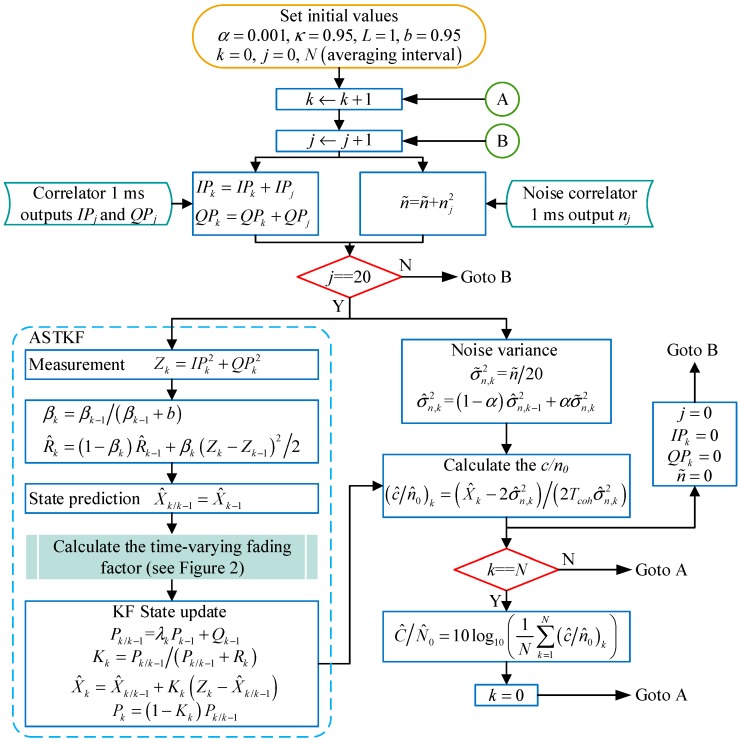
Summary of the adaptive strong tracking Kalman filter (ASTKF) carrier-to-noise ratio (*C/N_0_*) estimation algorithm.

A detailed algorithmic flow chart of the ASTKF *C/N_0_* estimation method is shown in Figure 3.

## 4. Computer Simulation Experiments

**Figure 4:**
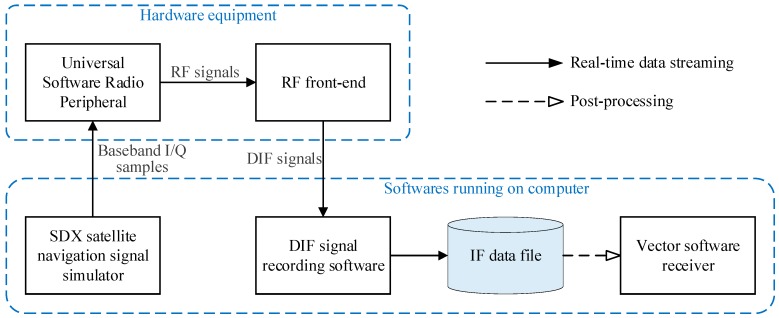
Simulation experiments data generation and processing flow.

**Figure 5:**
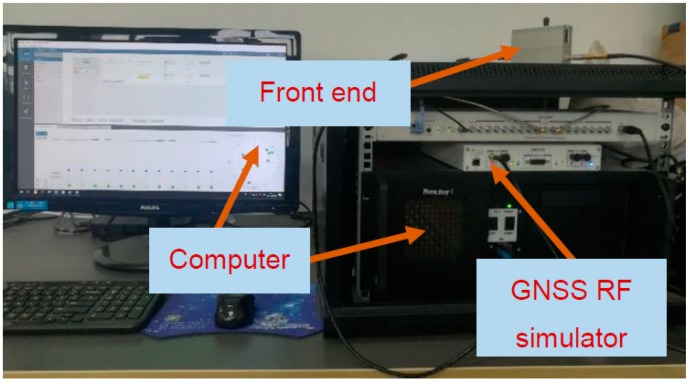
Global Positioning System (GPS) radio frequency (RF) signal simulation and digital intermediate frequency (IF) signal acquisition.

To evaluate the performance of the ASTKF *C/N_0_* estimation algorithm, simulation tests were conducted using the GNSS signal simulator and the vector software receiver. Figure 4 shows a block diagram of the simulation experiment’s data generation and processing. In the tests, the Skydel SDX satellite navigation signal simulator was used to generate in real-time the in-phase/quadrature (I/Q) samples representing the GPS baseband signals. Moreover, the SDX simulator can adjust the power of the simulation signal online [21]. Then, a universal software radio peripheral converted baseband I/Q samples to the GPS L1 C/A radio frequency (RF) signal. The RF front-end converted the simulated RF signal into a digital intermediate frequency (IF) signal and stored it on the computer. The signal simulation and acquisition equipment is shown in Figure 5. Table 2 presents the main parameters of the RF front end. The vector software receiver performed post-processing analysis on the digital IF signal and output the *C/N_0_* estimation results.

### 4.1. C/N_0_ Mutation Scenario Evaluation

The motion form of the receiver was set to static. In the simulation process, the signal *C/N_0_* was changed in step form to evaluate the estimation performance of the ASTKF *C/N_0_* estimator during *C/N_0_* hopping. At the beginning of the simulation, *C/N_0_* was set to 45 dB-Hz. At 60 s, *C/N_0_* was increased to 55 dB-Hz in steps. At 120 s, *C/N_0_* was reduced to 15 dB-Hz in steps, and at 180 s, it was restored to 45 dB-Hz.

To fairly evaluate the advantages of the strong tracking filter over the classical Kalman filter, a measurement noise adaptive technique that is based on the Allan variance is introduced into the standard amplitude Kalman filter. A *C/N_0_* estimation algorithm that is based on adaptive Kalman filter (AKF) is constructed as a benchmark. The averaging time for both methods is set to 0.5 s.

**Figure 6:**
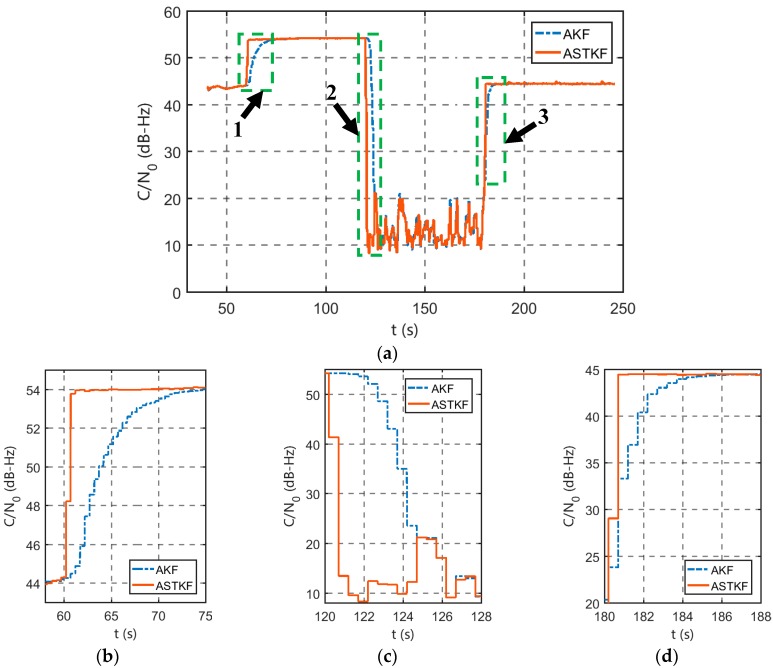
Comparison of the estimation results in a *C/N_0_* mutation environment (0.5 s averaging time): (**a**) a global figure; (**b**) a zoomed-in view of part 1; (**c**) a zoomed-in view of part 2; and (**d**) a zoomed-in view of part 3.

Figure 6 presents the *C/N_0_* estimation results of the AKF and ASTKF methods. The results of the AKF and ASTKF methods are approximately consistent when *C/N_0_* is stable. However, when *C/N_0_* shows a large jump (at 60 s, 120 s and 180 s), the ASTKF estimation method can track the *C/N_0_* change more quickly, while the AKF estimation result shows a significant lag.

When the signal amplitude changes abruptly, *C/N_0_* changes substantially. The system model that is represented by Equation (20) has a severe mismatch with the real model. The mean square error that is calculated by the classical Kalman filter in Table 1 is smaller than the true value, thereby resulting in a small filter gain value, and the impact of the measurement innovation on the state update is weak. According to Equation (22) and the calculation process of time-varying fading factor, we can see that the STKF utilizes the measurement innovation when calculating the one-step prediction mean square error matrix. Therefore, when *C/N_0_* jumps, the filter gain of the ASTKF method is larger than the filter gain of the AKF method, and the response to the sudden change in state is more rapid.

### 4.2. Algorithm Precision Analysis

To evaluate the estimation accuracy of the ASTKF estimation method, two sets of GPS L1 C/A signals that differed in terms of *C/N_0_* were generated by the SDX satellite signal simulator. For one set, *C/N_0_* was set to 55 dB-Hz, which corresponds to a strong satellite signal; for the other set, *C/N_0_* was set to 18 dB-Hz, which corresponds to a weak satellite signal. The digital IF signals were post-processed by the software receiver. Two classical methods, the NWPR and the VSM were selected as the reference. To analyse the effect of the averaging time, four averaging times (0.5 s, 1 s, 3 s and 5 s) were used.

In the simulation tests, the *C/N_0_* estimation results are smaller than the set values due to signal attenuation that is caused by the simulator, the RF front-end, and the connection cables. Therefore, the accuracy of the estimation is measured in terms of the standard deviation (Std) of the estimation results.

#### 4.2.1. Strong Signal Environment Evaluation

The *C/N_0_* of the simulated signal was set to 55 dB-Hz. In the real environment, the outdoor GPS signal has a *C/N_0_* of approximately 35~55 dB-Hz [17]. The *C/N_0_* of 55 dB-Hz is almost the maximum value of the real GPS signal, which represents a strong signal environment. The length of the sampled signal is approximately 10 minutes.

**Figure 7:**
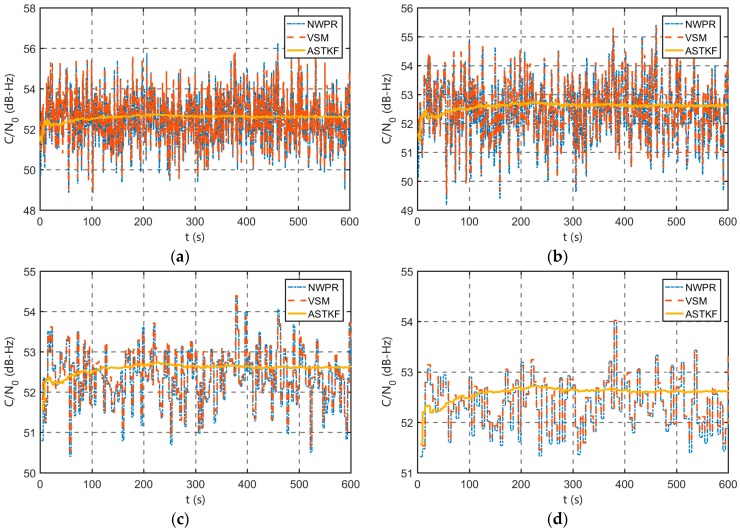
Simulation strong signal *C/N_0_* estimation results: (**a**) 0.5 s averaging time; (**b**) 1 s averaging time; (**c**) 3 s averaging time; and (**d**) 5 s averaging time.

Figure 7 presents the *C/N_0_* estimation results that were obtained using four averaging times. The estimation results of the two classical *C/N_0_* estimation methods are similar. In addition to the initial transition phase, the estimation results of the ASTKF method are more stable than those of the NWPR and the VSM.

Table 3 presents a statistical comparison of the estimation results. In the strong signal environment, the standard deviation of the ASTKF estimation results is smaller than that of the results of the other two methods using the same averaging time. In addition, according to Table 3, the estimated standard deviation of ASTKF using the averaging time of 0.5 s is smaller than that of NWPR and VSM using the averaging time of 5 s. Therefore, compared with the NWPR and the VSM, the ASTKF *C/N_0_* estimation algorithm can adopt a smaller averaging time, which facilitates the reduction of the lag of the estimation result. In the strong signal environment, using the ASTKF method, after the averaging time exceeds 0.5 s, continuing to increase the averaging time does not significantly improve the estimation accuracy of *C/N_0_*.

#### 4.2.2. Weak Signal Environment Evaluation

**Figure 8:**
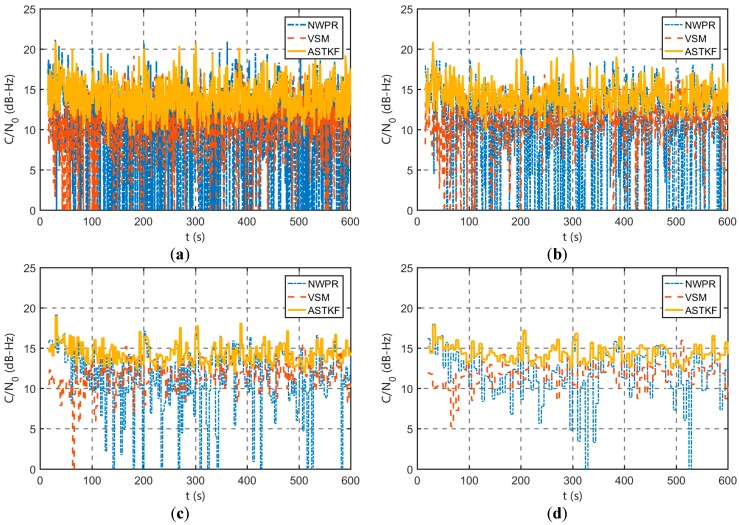
Simulation weak signal C/N0 estimation results: (**a**) 0.5 s averaging time; (**b**) 1 s averaging time; (**c**) 3 s averaging time; and (**d**) 5 s averaging time.

The simulation signal *C/N_0_* was set to 18 dB-Hz for the simulation of a weak signal environment with a simulation time of 10 min. Figure 8 presents the simulation results of the ASTKF *C/N_0_* estimation method in this weak signal environment. Table 4 presents a statistical comparison of the *C/N_0_* estimation results.

According to Figure 8 and Table 4, in the weak signal environment, the *C/N_0_* estimation error is larger than that in the strong signal environment. However, compared with the NWPR and the VSM, the estimation results of the ASTKF estimation method are more stable, which is consistent with the simulation results in the strong signal environment. Similarly, the estimated standard deviation of ASTKF using an averaging time of 0.5 s is smaller than that of NWPR with an averaging time of 5 s and that of VSM with an averaging time of 3 s. Therefore, regardless of the strength of the received satellite signal, the ASTKF estimation method can use a shorter averaging time than the NWPR and the VSM.

In addition, comparing Table 3 and Table 4, in the weak signal environment, increasing the averaging time can significantly improve *C/N_0_* estimation accuracy. Therefore, for receivers that are operating in special environments (such as indoor environments), the averaging time should be extended suitably.

## 5. Static Field Test

**Figure 9:**
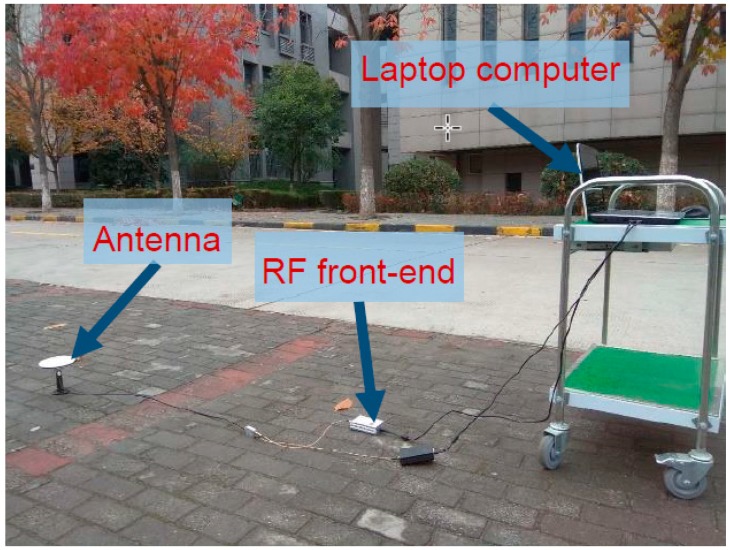
Static real signal acquisition environment.

**Figure 10:**
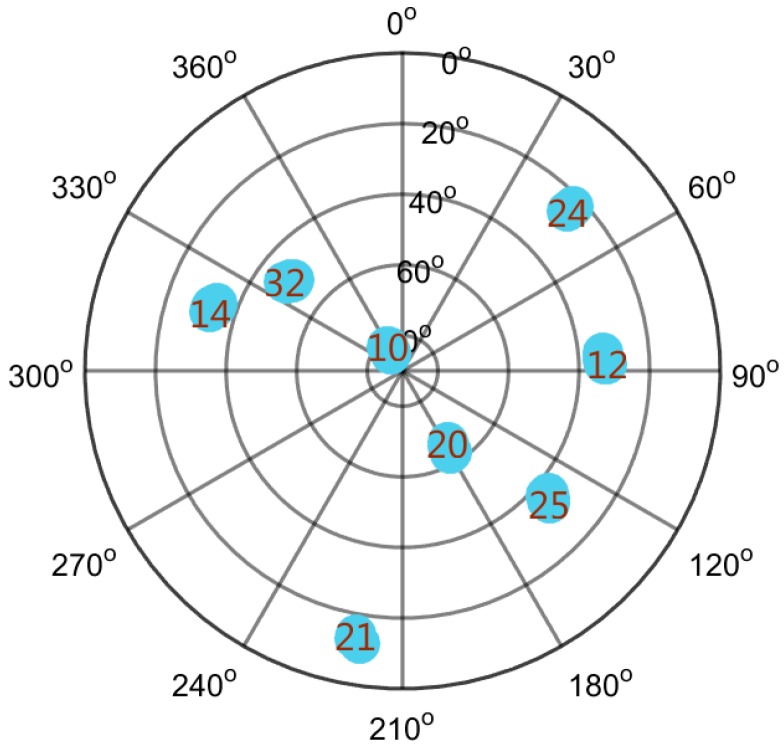
Sky plot of GPS satellites for the static field test.

To further evaluate the performance of the ASTKF method in a real environment, a static field test was conducted. The static test site was selected in an open, outdoor area, and the test environment is shown in Figure 9. In the test, the RF front-end was used to record the GPS L1 C/A code signal, and the signal length was approximately 5 min. The vector software receiver was used for post-processing analysis. During the field test, satellites 10, 12, 14, 20, 21, 24, 25 and 32 were visible. Figure 10 shows a sky plot of the visible GPS satellites. The ASTKF and two popular methods (NWPR and VSM) were used to estimate *C/N_0_* for comparative analysis. The averaging time of 0.5 s was selected as the empirical value of the averaging time.

**Figure 11:**
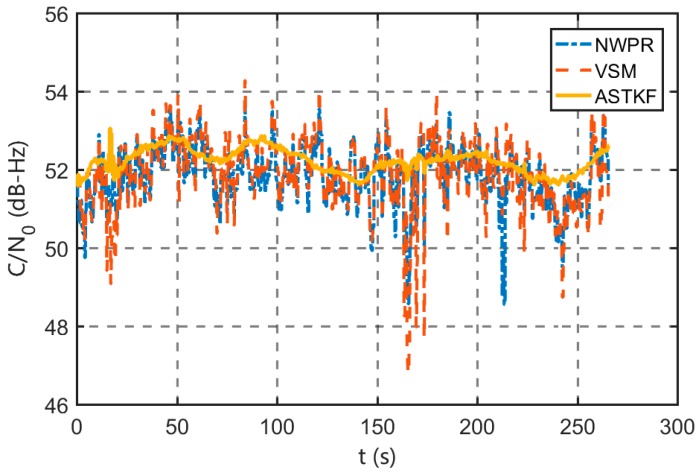
Satellite 10 *C/N_0_* (0.5 s averaging time).

**Figure 12:**
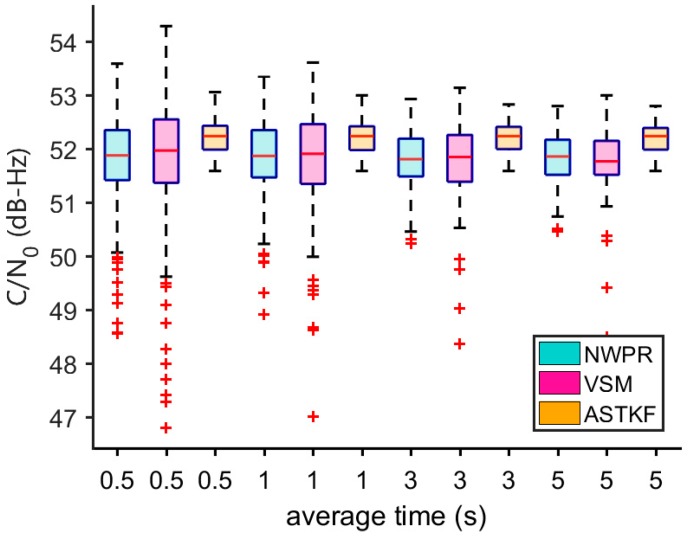
Satellite 10 *C/N_0_* estimation box plot comparison.

As can be seen from Figure 10, satellite 10 has the highest elevation angle, and the signal reception quality is satisfactory. In a short time (5 min), the signal power and *C/N_0_* do not change substantially. Figure 11 presents its *C/N_0_* estimation results. According to Figure 11, in the real signal environment, the ASTKF estimation results are less volatile than the NWPR and the VSM estimation results. Figure 12 is a box diagram of the *C/N_0_* estimation results of satellite 10 at various averaging times. According to Figure 12, the ASTKF method achieves higher accuracy (the result distribution is more concentrated). Moreover, the ASTKF estimation error with an averaging time of 0.5 s is less than that of the NWPR and the VSM with an averaging time of 5 s. These results are consistent with the conclusions of the simulation tests.

**Figure 13:**
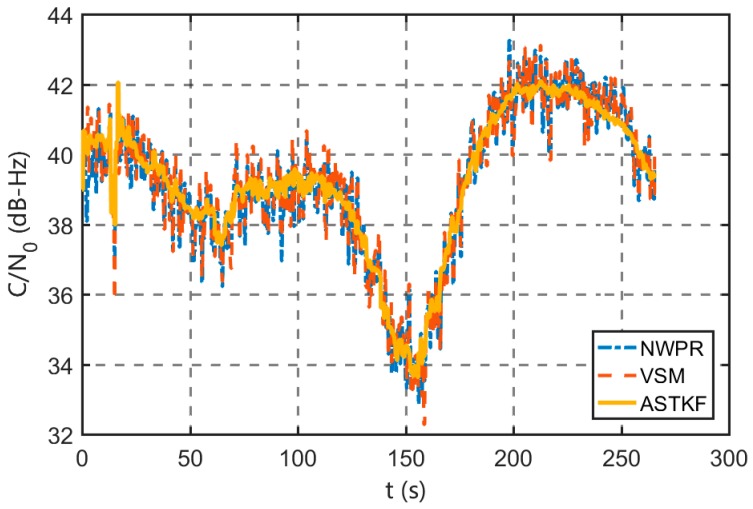
Satellite 24 *C/N_0_* (0.5 s averaging time).

As Figure 10 shows, satellite 24 has a low elevation angle, and the signal is susceptible to interference and attenuation. Satellite 24 is selected for evaluation of the estimation performance of the ASTKF method when the signal condition in the environment is poor. Figure 13 shows that the trends in the ASTKF and the other two methods are consistent. At 100~200 s, the signal power and *C/N_0_* show wide ranges of changes. The ASTKF method can correctly track this change in *C/N_0_*. The test results demonstrate that in the real environment, the ASTKF method realizes satisfactory estimation performance on signals of poor quality.

## 6. Conclusions

In this paper, we propose a *C/N_0_* estimation method that is based on an adaptive strong tracking Kalman filter, which considers the characteristics and application environment of the vector receivers. Based on the STKF and a measurement noise adaptation that is based on Allan variance, the ASTKF *C/N_0_* estimation method estimates the *C/N_0_* values of vector receivers in signal strength hopping and weak signal environments. The results of the first simulation test demonstrate that the estimated delay of the ASTKF method is significantly smaller than that of the AKF method when the *C/N_0_* jumps. The results of the second simulation experiment demonstrate that the estimated standard deviation of the ASTKF method with an averaging time of 0.5 s is smaller than that of the NWPR and the VSM with a longer averaging time in both the strong signal and the weak signal simulation environments. Therefore, compared to the NWPR and the VSM, the ASTKF method can use a shorter averaging time to reduce the lag of the estimation results. The static field test data demonstrate the applicability of the ASTKF method to signals in practice, and the ASTKF method can track large variations in the true signal *C/N_0_*. In addition, the results of the field test support the conclusion of the simulation test that the estimated standard deviation of the ASTKF method is significantly smaller than that of the NWPR and the VSM.

## Figures and Tables

**Table 1:** Classical Kalman filter (CKF) process [19].

**System propagation**	1. State prediction ${\hat{X}}_{k/k-1}=\Phi_{k,k-1}{\hat{X}}_{k-1}$ 2. Covariance propagation $P_{k/k-1}=\Phi_{k,k-1}P_{k-1}\Phi_{k,k-1}^{T}+Q_{k-1}$
**Measurement update**	3. Kalman gain $K_{k}=P_{k/k-1}H_{k}^{T}{\left(H_{k}P_{k/k-1}H_{k}^{T}+R_{k}\right)}^{-1}$ 4. State update ${\hat{X}}_{k}={\hat{X}}_{k/k-1}+K_{k}\left(Z_{k}-H_{k}{\hat{X}}_{k/k-1}\right)$ 5. Covariance update $P_{k}=\left(I-K_{k}H_{k}\right)P_{k/k-1}$

**Table 2:** RF front-end main parameters.

Quantization Bit	Noise Figure	IF Frequency	Sampling Frequency
2 bits	2.8 dB	3.996 MHz	16.369 MHz

**Table 3:** Strong signal *C/N_0_* estimation statistics.

	0.5 s Averaging Time		1 s Averaging Time		3 s Averaging Time		5 s Averaging Time	
	Mean (dB-Hz)	Std (dB-Hz)	Mean (dB-Hz)	Std (dB-Hz)	Mean (dB-Hz)	Std (dB-Hz)	Mean (dB-Hz)	Std (dB-Hz)
**NWPR**	52.47	1.22	52.42	1.05	52.35	0.71	52.32	0.52
**VSM**	52.54	1.17	52.49	1.00	52.42	0.67	52.40	0.50
**ASTKF**	52.58	0.15	52.58	0.15	52.57	0.14	52.57	0.14

**Table 4:** Weak signal *C/N_0_* estimation statistics.

	0.5 s Averaging Time		1 s Averaging Time		3 s Averaging Time		5 s Averaging Time	
	Mean (dB-Hz)	Std (dB-Hz)	Mean (dB-Hz)	Std (dB-Hz)	Mean (dB-Hz)	Std (dB-Hz)	Mean (dB-Hz)	Std (dB-Hz)
**NWPR**	9.88	6.22	10.30	5.69	11.19	4.30	11.71	3.20
**VSM**	10.51	4.42	10.93	3.57	11.64	2.18	11.90	1.58
**ASTKF**	13.94	2.03	14.09	1.71	14.25	1.29	14.32	1.07
